# Validity of Interviewer-Administered 24-h Dietary Recalls in Older Korean Women: A Pilot Study

**DOI:** 10.3390/nu15071757

**Published:** 2023-04-04

**Authors:** Seunghee Kim, Clara Y. Park

**Affiliations:** Department of Food and Nutrition, Chonnam National University, Gwangju 61186, Republic of Korea

**Keywords:** dietary assessment, 24-h recall, validity, Koreans

## Abstract

Despite the wide use of 24-h dietary recalls and differences in food culture between Western and Asian countries, limited studies validating 24-h dietary recalls have been performed in rice-based meals and in Asians. To determine the accuracy of 24-h dietary recalls in Koreans, 22 older women participating in a controlled-feeding study completed a single interviewer-administered 24-h dietary recall. The recalls of food items were classified as matches, exclusions, or intrusions. Portion size reports were categorized as corresponding (≤10% error), overreport, underreport, and missing. Recall accuracy was analyzed according to the type of dish, food group, or nutrients and compared by one-way analysis of variance or paired *t*-test. Participants reported 95% of the foods that they consumed. Sauces were most frequently missing. Corresponding portion sizes were 24%, while 43% were underreported. Kimchi was most frequently underreported. No difference was found among food groups. The recalled intakes of energy and most nutrients were similar to the actual intakes, with the exception of fat and sodium, which were underreported. The interviewer-administered 24-h dietary recall may be a reliable tool to assess food and nutrient intake in older Korean women. More accurate methods are necessary to assess sauce, kimchi, fat, and sodium intakes in the Korean diet.

## 1. Introduction

Interviewer-administered 24-h dietary recalls are used worldwide, including in national surveys such as the National Health and Nutrition Examination Survey (NHANES) [1]. Energy intake assessed by 24-h dietary recalls has been validated against direct observation [2], estimated energy of the provided diet [3], and the doubly labeled water (DLW) method [4,5]. In addition, the recall accuracy of macronutrient and micronutrient intakes has also been assessed [2,6]. The high recall accuracy reported in validation studies performed in the United States (US) and European countries indicates that the interviewer-administered 24-h diet recall is a reliable dietary assessment tool [2,3,4,5,6,7,8,9,10].

Although previous validation studies have shown a high accuracy of 24-h dietary recalls, the results may not be applicable to reports of Asian-style meals, as most studies have been performed in Western diets [3,4,5,6,7,8,9,10]. The composition of Korean meals differs from that of Western meals, as they consist of rice, soup, kimchi, and various side dishes (banchans). In addition, the ingredients, spices, and recipes used in Korean meals differ from those used in Western meals. Furthermore, Korea also conducts large nutrition surveys using the 24-h diet recall, such as the Korea National Health and Nutrition Examination Survey (KNHANES) and Korea Genome and Epidemiology Study. Some studies have assessed the relative validity of food frequency questionnaires against 24-h dietary recalls in Koreans [11]. However, to our knowledge, only a few validation studies for 24-h dietary recalls have been performed in Koreans, of which the accuracy of reported energy and sodium intakes were assessed [12,13,14]. These studies required the participants to take photographs of the foods during meals, reducing the surprise element of retrospective 24-h recalls. Another study assessed the validity of self-administered food records completed 24 h after intake in young Korean women in a controlled-feeding study [15]. The high inaccuracy of portion size estimates in this study may be due to the lack of assistance from food guides/models or interviewer. In addition, although the accuracy of 24-h recalls for nutrients may be important, its meaningfulness has recently been challenged [16,17]. As dietary patterns have become a large area of interest, understanding the reporting accuracy of foods is essential. Hence, there is a great need for validation studies of food and nutrient intake from 24-h dietary recalls in Koreans and rice-based meals.

The objectives of this study are to assess the accuracy of interviewer-administered 24-h dietary recalls in older Korean women participating in a controlled-feeding trial according to the food items consumed, portion size estimates, and nutrient intake. In this pilot study, we also investigated whether the recall accuracy differed according to the food group or type of dish (rice, soup, kimchi, banchans, sauce, snacks, and beverages).

## 2. Materials and Methods

### 2.1. Study Design

This work was a secondary study conducted among women to assess the effect of marine healing and sea mustard (*Undaria pinnatifida*) intake on menopausal symptoms [18] and the gut microbiome. Korean women aged 45–65 years participated in a 5-day controlled-feeding study during 8–12 July 2019 at Wando-gun, Jeollanam-do, South Korea. During the study, participants were provided with housing, meals, and snacks. All participants participated in preplanned activities. All meals, snacks, and activity participation were monitored for compliance. Participants were randomly selected on one of 4 days (days 2–5 of the study) of the controlled-feeding period to be interviewed for the 24-h recall. Participants were informed that a 24-h dietary recall would be administered; however, the date was not specified. The researchers did not mention any aspect regarding taking photographs of the foods to the participants in order to mimic a free-living setting. The reported food items and portion sizes were compared with the actual intakes according to the food group or type of dish. The reported and provided nutrients were analyzed by Computer Aided Nutritional analysis program for Professionals 5.0 (CAN-Pro 5.0; Korean Nutrition Society). The parent study was conducted according to the guidelines laid down in the Declaration of Helsinki, and all procedures involving human subjects were approved by the Institutional Review Board of Chonnam National University (IRB number: 1040198-190408-HR-020-07). Written informed consent was obtained from all subjects. The parent trial was registered at the Clinical Research Information Service (www.cris.cdc.go.kr; registration number: KCT0004025).

### 2.2. Participants

Based on the primary hypothesis of the parent study, women with menopausal symptoms who agreed to adhere to the controlled-feeding protocol were eligible to participate. Participants were recruited through posters and social networking services in June 2019. The sample size was determined by the feasibility of recruitment. The minimally detectable effect size with 22 participants for reported nutrient intake differs by nutrient (energy: 148 kcal; carbohydrate: 23 g; protein: 11 g; fat: 11 g; sodium: 373 mg, etc). Women who did not have menopausal symptoms, were administered antibiotics within the last 6 months, were smokers, had severe gastrointestinal diseases, or did not comply with the controlled-feeding protocol were excluded. Height and weight were measured with a stadiometer and scale, respectively; these measurements were then used to calculate body mass index (BMI). Data regarding age, residence, monthly family income, education, working status, and physical activity were collected using a questionnaire. Most women resided in Gwangju, some (*n* = 7) in the Jeollanam-do province, and one in the Gyeonggi-do province.

### 2.3. Diet

Participants were provided with 3 meals and 2–3 snacks per day for 5 days. Diets were provided according to the participants’ energy needs calculated according to the 2015 Dietary Reference Intakes for Koreans [19] or the mean nutrient intake of Koreans. Typical Korean-style meals, which consist of cooked rice, soup, kimchi, and 2–3 banchans, and snacks, were served on most days (Table 1). Most provided meals were similar to those recommended by the 2015 Dietary Guidelines for Koreans (rice, soup, kimchi, and 2–3 banchans) [19] and consisted of foods typically consumed by Koreans. Breakfast was simplified on some days due to traveling (days 1 and 2). For simple breakfasts, rice cakes were offered as they are enjoyed by most Koreans, especially older adults. Participants were asked only to consume the food provided, and all the food items provided had to be consumed.

All the ingredients were measured to the nearest 0.1 g during preparation. Foods were measured to the nearest 1 g before serving. Participants were provided meals on a food tray. Participants were not provided with written menus, and foods were not labeled with its name. The research staff monitored the intake of all meals and snacks throughout mealtime for compliance. One research staff member sat at a rectangular six-seat meal table and monitored up to 5 participants (participants were seated at each side of, across from, and diagonal from the research staff). One research staff member monitored the trashcan where the food trays were returned to ensure food was not thrown away. Based on the primary aims of the study, participants were randomized to the sea mustard or control group according to a computer-generated sequence. Participants randomized to the sea mustard group consumed meals in which sea mustard was added or used to substitute other vegetables in the control meal. Supplement use was not allowed.

### 2.4. Interviewer-Administered 24-h Dietary Recall

Participants were randomly selected and interviewed face-to-face before breakfast in private locations. Two trained dietitians conducted the interviews according to the Multiple-Pass Method (MPM) [20], which was modified to adapt some protocols of the KNHANES [21] and NHANES [1]. The MPM included the following steps: (1) A quick list associated with the day’s events, (2) Forgotten foods, (3) Time and occasion, (4) A detailed cycle, (5) Final probing [22]. The forgotten foods step was adapted to the Korean food culture to include snacks (such as fruit, bread, rice cakes, beverages [milk, soymilk, juice, and soft drinks], chocolate, candy, jelly, pie, and chips) and condiments (such as soy sauce, sesame seeds, and red pepper paste) that are frequently consumed by Koreans [21]. Additionally, a picture of a food tray with rice, soup, kimchi, and banchans was shown to the participants as per the protocol of the KNHANES [21]. Food models (rice and soup) and measuring guides, such as measuring cups, bean bags, concentric circles, rectangular grids, and rulers, were used according to the protocol of the NHANES [23]. Identical questionnaires, interview guides, food models, and measuring guides were provided to each interviewer. Each interviewer interviewed 11 participants within the 4 days (2–3 participants/d/interviewer). The interviews were not audiotaped.

### 2.5. Recall Accuracy of Food Items and Portions Sizes

We assessed the accuracy of the food items consumed and portion sizes reported by the participants by comparing them to the actual intake of food items and portion sizes. Using previously established definitions [16,17], we determined whether the reported food items matched those provided (matches). Exclusions (i.e., items consumed but not reported) and intrusions (i.e., items reported but not consumed) were also identified. Matches were additionally categorized as exact, close, or far matches, following the protocol of Kirkpatrick et al. [2]. For example, when soybean paste soup with spinach was consumed, a report of soybean paste soup with spinach was considered an exact match, whereas soybean soup with a different green vegetable (within the same food group) was considered a close match. An example of a far match is soybean soup with tofu. Classifications were reviewed and discussed by 2–3 research personnel. The portion size was determined by the sum of the ingredients in the food. The recall accuracy of portion sizes was determined as corresponding (90–110% of the actual intake), underreport (<90% of actual intake), and overreport (>110% of actual intake) of matched food items. Foods reported as volume were converted to weight using the Food Portion/Weight Database developed by the Ministry of Health and Welfare and the Korea Health Industry Development Institute [24]. If the participant reported unquantifiable amounts (i.e., “a little”, “a pinch”, “lightly seasoned”, etc.), the amount was considered missing.

Foods were categorized according to the type of dish (rice, soup, kimchi, banchans, sauces, snacks, and beverages) or food group (grains; protein foods [meat, poultry, fish, eggs, beans and legumes]; vegetables; fruits; dairy; and fats and sweets) [19]. The breakfast foods of days 1 and 2 (rice cakes, vegetable sticks, and cherry tomatoes) were considered snacks according to the traditional Korean meal setting. Sauces mostly consisted of spices, soy sauce, oil, and fermented (soybean or red chili) paste; therefore, they were excluded from the food group analysis. Soups were placed in the food group category based on their solid ingredients.

### 2.6. Recall Accuracy of Nutrient Intake

All reported diets and actual recipes were matched to food codes in the widely used nutrient analysis software CAN-Pro 5.0 and analyzed for energy, carbohydrate, protein, fat, calcium, sodium, potassium, iron, folate, cholesterol, fiber, phosphorus, zinc, vitamin A, retinol, β-carotene, vitamin E, vitamin C, thiamin, riboflavin, niacin, and vitamin B6 content. The difference between reported and actual energy intake per item was calculated.

### 2.7. Statistical Analyses

The accuracy of each participant’s food items and the portion size was calculated before analyzing the mean of the total study population. The percentage of matches and exclusions are reported as means, similar to that in the study by Kirkpatrick et al. [2]. Comparisons among the types of dishes or food groups were assessed using a one-way analysis of variance and the Tukey–Kramer post hoc test. The mean kilocalorie inaccuracy among types of dishes or food groups was assessed. The actual total energy and nutrient intakes during days 1–4 were compared with reported intakes (investigated on days 2–5) using the paired *t*-test or the Wilcoxon signed-rank test (presented as means ± standard deviation [SD]). Bland–Altman plots were constructed to visualize the agreement between the reported energy intake and actual energy intake. All statistical analyses were performed using SAS version 9.4 (SAS Institute Inc., Cary, NC, USA). The null hypotheses were rejected when *p* < 0.05.

## 3. Results

### 3.1. Participant Characteristics

Twenty-two women participated in the study (Table 2). The mean (±SD) age of the participants was 55.5 (±5.2) years, and the mean BMI was 23.1 (±3.7) kg/m^2^. Most participants (86.4%) were high school graduates or had a higher education status.

### 3.2. Accuracy of Food Items Reported

Approximately 20 food items were served per study day (Table 3). Participants reported over 95% of the consumed foods. When assessed by type of dish, the proportion of exact, close and far matches were 68.0%, 22.2%, and 4.9%, respectively. The proportion of exact and close matches added up to ≥ 90% regardless of dish type, except for sauces (81.8%). Sauces also had a low rate of exact matches (34.1%).

Regarding food groups, all food groups had a match rate of ≥97%, with no significant difference among the groups when all matches were considered (Table 4). The proportion of exact matches of the fruit group and fats and sweets group (97.0% and 100%, respectively) was higher than that of the grains and dairy groups (70.9% and 68.2%, respectively). The proportion of matches and exclusions and the number of intrusions did not differ according to the interviewer or interview date.

### 3.3. Accuracy of Portion Size Estimates

When analyzed by type of dish, the proportion of corresponding, overreport and underreport of portion sizes were 24%, 26%, and 43%, respectively (Table 3). Participants reported beverage portion size most accurately (corresponding: 65.9% and underreport: 6.8%). Seventy-two percent of kimchi portion sizes were underreported. However, the inaccuracy in the reported kilocalories was not different among types of dishes (Table S1). Despite the high missing rate of sauces (25.0%), this only accounted for 13 kcals per item. Regarding food groups, the dairy group had the highest rate of corresponding matches (77.3%), while only 6.4% and 8.8% of protein foods and vegetables were corresponding matches, respectively (Table 4). However, the mean total kilocalorie inaccuracy did not differ among food groups (Table S2). The rates of overreporting and underreporting within each food group were similar for most food groups. The accuracy of the portion size report did not differ between interviewers; however, a trend (*p* = 0.06) in the increase of corresponding matches was observed with time.

### 3.4. Comparison of Actual and Reported Intakes for Energy and Nutrients

**Figure 1:**
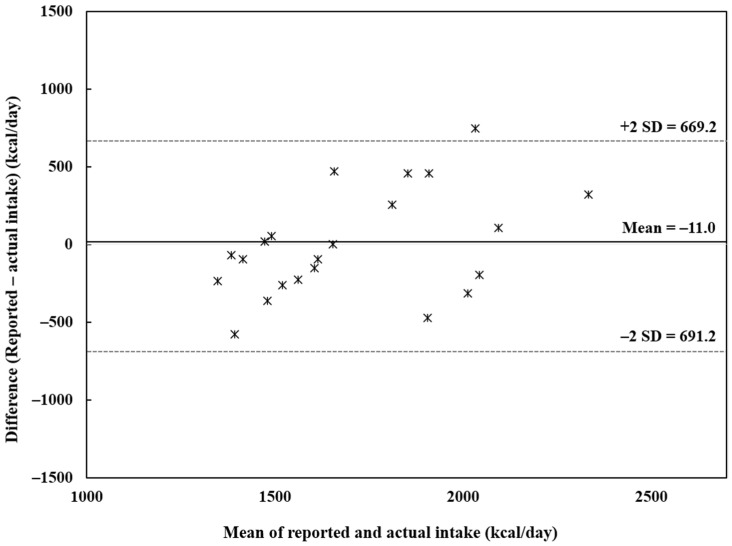
Bland–Altman plot for the reported and actual energy intake among older Korean women (*n* = 22) Symbols represent each individual. SD: standard deviation.

When analyzed using CAN-Pro, the actual and reported intakes for energy, and most nutrients, did not differ (Table 5 and Table S3). However, the fat and sodium intakes were underreported (mean differences: fat = −10.7 g [95% CI: −16.3 g, −4.99 g] and sodium = −1270 mg [95% CI: −1655 mg, −909 mg]). The Bland–Altman plot for energy shows that only one participant had >2 SD difference between reported and actual intake (Figure 1).

## 4. Discussion

The 24-h dietary recall procedure administered to healthy older Korean women elicited relatively accurate reports of intakes of food items, portion sizes, and nutrients. The participants’ recall accuracy of food items did not differ according to the food group or type of dish, except for sauces, which were frequently forgotten. Portion sizes were relatively accurately reported for beverages, while kimchi portion sizes were frequently underreported. Energy and most nutrients were accurately recalled; however, fat and sodium intakes were underestimated by the 24-h dietary recall.

Although the validity of energy in foods reported in 24-h dietary recalls has been assessed in Koreans, this is the first study that assessed the validity of 24-h dietary recalls of food and nutrient intake in Koreans. The validity of 24-h dietary recalls has been tested in numerous studies on Western diets; however, the findings may not be applicable to Asian diets as the food culture in Asian diets is different from that in Western diets. Asian meals typically consist of rice, soup, and several banchans. One study assessed the validity of 24-h dietary recalls in Chinese–American adolescents; however, only one meal was served with rice [25]. A few studies performed in Koreans assessed the accuracy of reported energy and sodium intakes [12,13,14]. However, participants in these studies mandatorily photographed or recorded their intake the day before the interview. Therefore, participants of these studies are more likely to remember their intakes better during the previous day, compared to 24-h dietary recalls administered unexpectedly. Participants of the current study were allowed, but not encouraged, to take pictures of their meals, similar to individuals in a free-living setting. None of the participants used photographs of the foods during the interview. We did not provide menus or label food to maintain the setting as similar to free-living conditions as possible, as menus or labels may enhance the participants’ memory [26]. The present study is a meaningful contribution to the literature, as to the best of our knowledge, this is the first study to validate 24-h dietary recalls for food and nutrient intakes for rice-based meals and among Korean adults.

The 24-h dietary recall was relatively accurate in estimating the intake of food items and portion sizes, regardless of the food group and type of dish, except sauces and kimchi. Sauces had a low rate of exact matches and a high rate of exclusions. Condiments, such as mayonnaise and cheese, have been reported to be frequently omitted by US adults during recalls, which may lead to a lower recall accuracy of fat intake than the actual intake, as assessed by the Automated MPM and Automated Self-Administered 24-h (ASA24) Dietary Recall methods [2]. The sauces and spreads consumed during this study were mostly made in-house and consisted of soy sauce, red pepper sauce, or soybean curd (all high in sodium) mixed with condiments such as sesame seed oil, sesame seeds (both high in fat), garlic, green onions, and red pepper powder. Therefore, the omission or underreporting of these sauces would have affected the reported nutrient intake, especially sodium and fat. In addition, the high proportion of underreporting of kimchi—also high in sodium—may have contributed to the underreporting of sodium intake. As the quantity of sauce consumed and the calorie density of kimchi are both relatively low, the inaccurate recalls did not affect the accuracy of the reported intake of total energy or other nutrients. On the other hand, many studies have reported that snacks and desserts are frequently omitted or underreported [27,28]. However, our participants’ recall accuracy for snacks and drinks was not different from that for other types of dishes. This may be due to the controlled-feeding setting or because snacks were mostly provided immediately before or after activities, making them easier to remember with the use of the multiple-pass method. Similarly, Kirkpatrick et al. [2] did not find a high omission rate for sweets, snacks, and desserts, possibly due to the study design that involved providing such foods with meals instead of providing them between meals. Our results suggest that 24-h dietary recalls are reliable tools for the assessment of food and nutrient intake; however, better interview protocols adapted to the Korean food culture are needed for accurate estimations of sauce and kimchi, sodium, and fat intakes.

In line with the accurate recalls of food items and portion size, the reported energy intake from the 24-h dietary recalls was similar to the actual energy intake. Our results differ from those reported by recent studies in older Korean men and women that compared the energy intake reported using the 24-h dietary recall method with energy expenditure assessed using the DLW method [12,13]. Despite the participants mandatorily taking pictures or recording all foods consumed on the day before the recall in previous studies, men tended to underreport energy intake by approximately 4.6% (*p* = 0.069), while women underreported 534 kcal/day of energy intake (25.9% of total energy expenditure assessed using the DLW method; *p* < 0.001). Participants of the present study were aware of the upcoming interview as we could not interview all participants in one day owing to limited resources. Although participants were not aware of which day the interview would be performed, those interviewed later in the week may have anticipated the interview. In order to decrease error due to anticipation, we planned the interview to end a day earlier than the controlled feeding study; thus, the last participants were interviewed with 2 days in view. Still, we observed a slight increase in accuracy with time. Participants of large surveys such as NHANES and KNHANES may also anticipate an upcoming interview as food models or portion size guides are distributed, or telephone interviews are scheduled [1,21]. The high accuracy of our participants may also be because the participants in our study were younger than those in other studies. These characteristics may have increased the ability of our participants to recall specific foods and ingredients accurately compared with that of other older participants included in other studies. In addition, methodological errors of the DLW method are independent of those of CAN-Pro, which may have increased the gap between these two methods in previous studies. As we used CAN-Pro to analyze the energy intake reported using the 24-h dietary recall and the actual intake, our study may have overlooked the possible systematic error. However, using CAN-Pro for both analyses may be more effective in assessing recall bias. Further studies including other age groups and larger sample sizes are required to assess the validity of 24-h dietary recalls for assessing energy intake.

This study has some limitations. First, the limited selection of foods during the study due to the controlled-feeding design and use of individual trays may limit the generalizability of the results to free-living individuals. In addition, using a picture of a food tray, rather than a picture of a common home or restaurant meal table-setting, may have increased the recall accuracy. Individual trays are frequently used in cafeterias at schools, workplaces, hospitals, nursing homes, and the military in Korea. However, in homes or restaurants, most banchans are traditionally shared rather than served individually. This food-sharing culture may challenge the interviewees’ portion size estimations and validation method of 24-h recalls. On the other hand, after experiencing the COVID-19 pandemic, more families and restaurants are using individual plates instead of sharing food directly from the dish. The present study using a controlled-feeding study design offers a unique opportunity to assess the accuracy of dietary recalls as the food and ingredient intakes were known to the researchers, and potential biases associated with observers could be eliminated. Second, the small sample size and participants’ experience and knowledge of cooking may limit the generalizability of our results. Previous studies indicate reporting bias according to BMI [4,9,29]. However, the small sample size and narrow range of BMI did not enable us to assess differences by BMI. Due to the primary hypothesis of the parent study, adults interested in health-related behaviors, including food, may have been more motivated to participate. In addition, most participants were responsible for meal preparation at home and may be more interested in and capable of predicting the type and amount of ingredients in mixed dishes by taste. Future studies that include men and participants of various age groups and health conditions are required. Finally, although the date was not specified, participants knew of the upcoming 24-h dietary recall. Despite these limitations, this is the first validation study for 24-h dietary recalls of food and nutrient intakes among older Korean females.

The interviewer-administered 24-h dietary recall may be a valid tool to measure food and nutrient intakes in older Korean women. With the exception of sauces and kimchi, women reported food items accurately regardless of the type of dish or food group. The reported intakes of most nutrients were similar to the actual intakes; however, the interview protocol may require improvements for the assessment of fat and sodium intakes.

## 5. Conclusions

Interviewer-administered 24-h dietary recall may be a valid tool to measure food and nutrient intakes in older Korean women when served individually. With the exception of sauces and kimchi, women reported food items accurately regardless of the type of dish or food group. The reported intakes of most nutrients were similar to the actual intakes; however, the interview protocol may require improvements for the assessment of fat and sodium intakes. Further validation studies with a greater sample size designed for participants to consume foods freely are required.

## Figures and Tables

**Table 1:** The food items served during the study.

Meal	Day 1	Day 2	Day 3	Day 4
Breakfast	- Steamed white rice cake - Honey-filled rice cake - Apple juice	- Steamed sweet pumpkin rice cake - Cucumber sticks - Carrot sticks - Cherry tomatoes - Orange juice	- Rice - Soybean paste soup with tofu and squid - Seasoned chives - Acorn jelly & soy sauce - Kimchi	- Vegetable (or seaweed) rice porridge - Soy sauce braised quail eggs - Seasoned spinach - Kimchi - Cherry tomatoes
Lunch	- Soybean sprout rice and soy sauce - Beef and radish soup - Steamed eggs - Young summer radish seasoned with soybean paste - Kimchi	- Bibimbap (with seaweed) and red pepper paste - Soybean paste soup with spinach - Korean-style barbequed pork (bulgogi) - Fried egg - Kimchi	- Rice - Dried pollack soup with radish - Stir-fried squid with bean sprouts - Seasoned cucumber - Kimchi	- Rice - Dried pollack soup with radish (or seaweed) - Smoked duck with chives - Seasoned chive and onions - Grilled garlic - Spicy seasoned radish - Kimchi
Afternoon snack	- Watermelon - Yogurt	- Biscuits - Sweet rice punch (shikhye)	- Biscuits - Sweet rice punch (shikhye)	- Morning roll - Strawberry jam - Yogurt drink
Dinner	- Dough flake soup (sujebi) with perilla seed powder (and seaweed) - Beef rice ball - Seasoned smoked oyster with vegetables - Seasoned spinach - Kimchi	- Rice - Soybean paste soup with mushrooms and perilla seed powder - Boiled pork - Soybean paste and red pepper paste mixture (with seaweed powder) (ssamjang) - Lettuce (or seaweed) - Cucumber sticks - Kimchi	- Rice - Kimchi stew (kimchi-jjigae) - Korean-style barbequed beef (bulgogi) - Stir-fried mushroom with perilla seed powder - Lettuce and perilla leaves (or seaweed) - Kimchi	- Rice - Soybean paste soup with young summer radish (or seaweed) - Stir-fried pork with kimchi - Steamed tofu - Cucumber chili pepper seasoned with soybean paste
Evening snack	- Plum	- Banana - Yogurt	- Watermelon - Yogurt	- Boiled potatoes - Oriental melon

**Table 2:** Characteristics of participants in the 24-h dietary recall validation study (*n* = 22).

Variables	Mean ± SD or *n* (%)
Age (years)	55.5 ± 5.2
BMI (kg/m^2^)	23.1 ± 3.7
Residence	
City	16 (72.7)
Rural areas	6 (27.3)
Monthly family income (KRW) ^1^	
<2,000,000	2 (9.1)
2,000,000–4,000,000	9 (40.9)
>4,000,000	9 (40.9)
Education	
Middle school graduate	3 (13.6)
High school graduate	10 (45.5)
≥College graduate	9 (40.9)
Employed	6 (27.3)
Physically active	11 (50.0)

BMI: body mass index; SD: standard deviation; ^1^ Two participants were missing household income data.

**Table 3:** Accuracy of food items and portion size of 24-h diet recalls in relation to actual intake according to the type of dish among older Korean women (*n* = 22) ^1^.

Type of Dish	Mean Number of Items Served	Accuracy of Food Items						Accuracy of Portion Size			
		Matches (%)				Exclusions	Intrusions ^2^	Corresponding (%)	Overreport (%)	Underreport (%)	Missing (%)
		Total	Exact	Close	Far	(%)					
Rice	2.5	100 ^a^	67.4 ^a^	30.3 ^a^	2.3 ^a^	0 ^a^	0	28.0 ^a^	40.9	31.1 ^ab^	0.0 ^a^
Soup	2.3	97.7 ^a^	59.1 ^ab^	34.8 ^a^	3.8 ^ab^	2.3 ^a^	0	12.1 ^a^	27.3	58.3 ^ac^	2.3 ^a^
Kimchi	2.3	95.5 ^ab^	90.9 ^a^	0 ^b^	4.5 ^ab^	4.5 ^ab^	1	8.3 ^a^	15.2	72.0 ^c^	4.5 ^a^
Banchans	6.0	95.6 ^ab^	73.5 ^a^	17.8 ^ab^	4.3 ^ab^	4.4 ^ab^	1	6.5 ^a^	31.7	57.4 ^ac^	4.4 ^a^
Sauce	1.5	81.8 ^b^	34.1 ^b^	29.5 ^a^	18.2 ^b^	18.2 ^b^	0	15.9 ^a^	18.2	40.9 ^a^	25.0 ^b^
Snacks	4.7	99.4 ^ab^	84.8 ^a^	13.0 ^ab^	1.5 ^a^	0.6 ^a^	1	31.4 ^a^	33.3	34.7 ^ab^	0.6 ^a^
Beverages	1.2	95.5 ^ab^	65.9 ^a^	29.5 ^a^	0 ^a^	4.5 ^ab^	0	65.9 ^b^	18.2	6.8 ^b^	9.1 ^ab^
All foods	20.4	95.1	68.0	22.2	4.9	4.9	3	24.0	26.4	43.0	6.6

^1^ Reported portion sizes were categorized according to the error of the recalled amount of food intake: corresponding (≤10% error), overreport (>110% of actual intake), underreport (<90% of actual intake), and missing. Statistical analysis was performed using ANOVA and the Tukey–Kramer post hoc test. Means within a column with unlike superscript letters were significantly different (*p* < 0.05). ^2^ Total number of intrusions for all participants.

**Table 4:** Accuracy of food items and portion size of 24-h diet recalls in relation to actual intake according to the food group among older Korean women (*n* = 22) ^1^.

Food Group	Mean Number of Items Served	Accuracy of Food Items						Accuracy of Portion Size			
		Matches (%)				Exclusions (%)	Intrusions ^2^	Corresponding (%)	Overreport (%)	Underreport (%)	Missing (%)
		Total	Exact	Close	Far						
Grains	4.8	97.0	70.9 ^a^	23.2 ^a^	3.0 ^ab^	3.0	0	24.5 ^ab^	35.5 ^ab^	37.0 ^ab^	3.0
Protein foods	2.7	97.0	80.3 ^abc^	15.2 ^ab^	1.5 ^ac^	3.0	1	6.4 ^b^	49.6 ^a^	40.9 ^ab^	3.0
Vegetables	9.0	97.3	75.4 ^ab^	14.9 ^ab^	7.1 ^a^	2.6	2	8.8 ^b^	21.8 ^ab^	64.7 ^b^	4.7
Fruits	1.8	98.5	97.0 ^bc^	1.5 ^b^	0 ^bc^	1.5	0	25.8 ^ab^	37.9 ^ab^	33.3 ^ac^	3.0
Dairy	1.0	100	68.2 ^a^	31.8 ^a^	0 ^bc^	0	1	77.3 ^c^	18.2 ^b^	4.5 ^c^	0
Fats & sweets	0.7	100	100 ^c^	0 ^b^	0 ^bc^	0	0	43.8 ^a^	25.0 ^ab^	31.3 ^ac^	0
All foods ^3^	20.0	98.2	81.1	15.1	2.0	1.8	4	30.5	31.6	35.5	2.4

^1^ Reported portion sizes were categorized according to the error of the recalled amount of food intake: corresponding (≤10% error), overreport (>110% of actual intake), underreport (<90% of actual intake) and missing. Statistical analysis was performed using analysis of variance and the Tukey–Kramer post hoc test. Means within a column with unlike superscript letters were significantly different (*p* < 0.05). ^2^ Total number of intrusions for all participants. ^3^ The number of all food items served differs from that in Table 3 due to the exclusion of sauces and the addition of sea mustard powder. The sauces mainly consisted of spices and did not belong to a specific food group. Sea mustard powder was added to the vegetable group in this analysis.

**Table 5:** Mean (±SD) values of served and reported intakes of energy and select nutrients among older Korean women (*n* = 22) ^1^.

Nutrient	Served Intake	Reported Intake	Mean Difference (95% CI)	*p* Value
Energy (kcal)	1715 ± 253	1704 ± 384	−11 (−162, 140)	0.88
Carbohydrate (g)	244.5 ± 30.0	256.5 ± 56.8	12.0 (−11.8, 35.7)	0.31
Protein (g)	78.2 ± 10.6	85.4 ± 24.4	7.2 (−3.4, 17.8)	0.17
Fat (g)	47.0 ± 21.6	36.3 ± 15.7	−10.7 (−16.3, −5.0)	0.001
Calcium (mg)	468.0 ± 49.6	433.8 ± 105.2	−34.2 (−83.1, 14.7)	0.16
Sodium (mg)	3271 ± 306	1989 ± 726	−1282 (−1655, −909)	<0.001
Potassium (mg)	3094 ± 615	3052 ± 793	−42 (−315, 230)	0.75
Iron (mg)	21.8 ± 7.5	25.2 ± 17.6	3.3 (−5.2, 11.9)	0.77
Folate (μg)	554 ± 164	511 ± 207	−43 (−101, 15)	0.14

SD: standard deviation; CI: confidence interval. ^1^ Nutrient intakes were analyzed using the Computer Aided Nutritional analysis program for Professionals 5.0 (Korean Nutrition Society). Paired *t*-test was used for normally distributed data, and the Wilcoxon signed-rank test was used for non-normally distributed data.

## Data Availability

The data presented in this study may be available on request from the corresponding author.

## Supplementary Materials

The following supporting information can be downloaded at: https://www.mdpi.com/article/10.3390/nu15071757/s1, Table S1: Mean difference between energy served and energy reported from 24-h recalls in kcals per item according to the type of dish among older Korean women (*n* = 22); Table S2: Reporting error of 24-h recalls in kcals per item according to food group in relation to actual intake among older Korean women (*n* = 22); Table S3: Mean (±SD) values of served and reported nutrient intakes among older Korean women (*n* = 22).
