# Supplementary material for: Validity of Interviewer-Administered 24-h Dietary Recalls in Older Korean Women: A Pilot Study
Source: Nutrients. 2023 Apr 4;15(7):1757. doi: 10.3390/nu15071757 (PMC10096839; doi:10.3390/nu15071757)
Supplement: Supplementary file 1 [file nutrients-15-01757-s001.zip › nutrients-2023451-SI.pdf]

Supplementary Materials

Table S1. Reporting error of 24-hour recalls in kcals per item according to the type of dish among older Korean women (*n* = 22) [1].

	**Total energy served (kcal)**	**Mean energy served (kcal/item)**	**Mean energy difference between served and reported intake (kcal/item)**				Missing (kcal/item)
			**All**	**Corresponding**	Overreport	Underreport	
Rice	539.7	221.7	44.5	9.5	85.5	-22.5	N/A
Soup	203.5	93.3	-22.4	-0.8	35.6	-49.7	-282.0[a]
Kimchi	17.8	8.1	-0.6	-0.4	13.2	-5.3	-6.9[b]
Banchans	499.6	79.2	17.1	-1.2	50.6	-29.8	-52.0[bc]
Sauce	23.5	17.3	-1.4	-0.2	9.4	-6.3	-13.0[b]
Snacks	336.5	73.1	10.3	2.0	36.3	-29.9	-68.3[bc]
Beverages	75.8	50.3	5.9	-0.6	37.1	-23.5	-90.0[c]
All foods	1696.5	83.1	7.2	1.8	41.3	-23.7	-45.1

N/A: not applicable (no missing report). [1] Reported portion sizes were categorized according to the error of the reported amount of food intake: corresponding (≤ 10% error), overreport (> 110% of actual intake), under report (< 90% of actual intake), and missing. Statistical analysis was performed using analysis of variance and the Tukey–Kramer post hoc test. Proportions of estimates in each column without a common superscript letter differ.

Table S2. Reporting error of 24-hour recalls in kcals per item according to food group in relation to actual intake among older Korean women (n = 22) [1].

	Total energy served (kcal)	Mean energy served (kcal/item)	Mean energy difference between served and reported intake (kcal/item)				Missing (kcal/item)
			Total	Corresponding	Overreport	Underreport	
**Grains**	**845.3**	**177.3**	42.9	7.8	64.7[a]	-20.3	-131.1
Protein foods	436.3	145.2	16.2	-1.9	78.4[a]	-69.9	-124.5
Vegetables	194.7	23.1	-1.0	-0.1	16.0[b]	-14.1	-13.4
Fruits	106.9	56.3	8.4	-3.7	39.3[ab]	-21.8	-90.0
Dairy	56.8	56.8	1.4	-1.2	17.4[ab]	-18.1	N/A
Fats and sweets	40.5	40.5	4.5	-0.1	31.4[ab]	-10.5	N/A
All foods[2]	1681.0	84.2	12.4	1.1	47.8	-28.5	-58.9

N/A: not applicable (no missing report). [1] Reported portion sizes were categorized according to the error of the recalled amount of food intake: corresponding (≤ 10% error), overreport (> 110% of actual intake), under report (< 90% of actual intake), and missing. Statistical analysis was performed using analysis of variance and the Tukey–Kramer post hoc test. Proportions of estimates in each column without a common superscript letter differ. [2] The total energy served differs from that in Supplementary table 1 due to the exclusion of sauces and addition of sea mustard powder. Sauces mostly consisted of spices and did not belong to a specific food group. Sea mustard powder was added to the vegetable group in this analysis.

Table S3. Mean (± SD) values of served and reported nutrient intakes among older Korean women (n = 22) [1].

Nutrient	Served intake	Reported intake	Mean difference (95% CI)	*P* value
Cholesterol (mg)	342.1 ± 37.3	382.8 ± 184.8	40.7 (-34.9, 116.2)	0.28
Fiber (g)	23.5 ± 3.8	21 ± 5.2	-2.50 (-4.96, -0.05)	0.046
Phosphorus (mg)	1067.9 ± 99.2	1098.8 ± 258.8	30.9 (-80.9, 142.7)	0.57
Zinc (mg)	12.1 ± 3.1	11.9 ± 2.6	-0.2 (-1.7, 1.4)	0.82
Vitamin A (μg RAE)	510 ± 189	577 ± 274	67 (-22, 155)	0.13
Retinol (μg)	128.3 ± 112.1	136.7 ± 117.5	8.4 (-19.7, 36.5)	0.04
β-carotene (μg)	4581 ± 1556	5279 ± 2977	698 (-303, 1699)	0.16
Vitamin E (mg)	10.7 ± 0.7	9.1 ± 2.1	-1.6 (-2.7, -0.5)	0.006
Vitamin C (mg)	101.5 ± 55.8	96.3 ± 66	-5.3 (-21.2, 10.7)	0.50
Thiamin (mg)	1.7 ± 0.4	1.7 ± 0.7	0.04 (-0.17, 0.24)	0.71
Riboflavin (mg)	1.4 ± 0.4	1.5 ± 0.5	0.06 (-0.12, 0.24)	0.52
Niacin (mg)	12.4 ± 2.3	13.3 ± 4.6	0.88 (-1.03, 2.78)	0.35
Vitamin B6 (mg)	1.6 ± 0.3	1.6 ± 0.4	0.04 (-0.17, 0.24)	0.71

CI: confidence interval; RAE: retinol activity equivalent. [1] Nutrient intakes were analyzed using Computer Aided. Nutritional analysis program for Professionals 5.0 (Korean Nutrition Society). The paired *t*-test was used for normally distributed data and the Wilcoxon signed-rank test was used for non-normally distributed data.
